# Does Stigmatized Social Risk Lead to Denialism? Results from a Survey Experiment on Race, Risk Perception, and Health Policy in the United States

**DOI:** 10.1371/journal.pone.0147219

**Published:** 2016-03-10

**Authors:** Yarrow Dunham, Evan S. Lieberman, Steven A. Snell

**Affiliations:** 1 Department of Psychology, Yale University, New Haven, Connecticut, United States of America; 2 Department of Political Science, Massachusetts Institute of Technology, Cambridge, Massachusetts, United States of America; 3 Social Science Research Institute, Duke University, Durham, North Carolina, United States of America; Loyola University Chicago, UNITED STATES

## Abstract

In this article, we report findings from an original survey experiment investigating the effects of different framings of disease threats on individual risk perceptions and policy priorities. We analyze responses from 1,946 white and African-American participants in a self-administered, web-based survey in the United States. We sought to investigate the effects of: 1) frames emphasizing disparities in the racial prevalence of disease and 2) frames emphasizing non-normative (blameworthy or stigmatized) behavioral risk factors. We find some evidence that when treated with the first frame, African-Americans are more likely to report higher risk of infection (compared to an African-American control group and to whites receiving the same treatment); and that whites are more likely to report trust in government data (compared to a White control group and to African-Americans receiving the same treatment). Notwithstanding, we find no support for our hypotheses concerning the interactive effects of providing both frames, which was a central motivation for our study. We argue that this may be due to very large differences in risk perception at baseline (which generate limits on possible treatment effects) and the fact that in the context of American race relations, it may not be possible to fully differentiate racialized and stigmatized frames.

## 1 Introduction

One of the most important welfare-preserving functions that government officials, international organizations, and other governance actors can perform is the provision of information about possible dangers and threats to citizens. Such information can be used by citizens to take actions to protect themselves from such dangers and possibly to support policies that would help to mitigate the consequences of those risks. Because public officials generally cannot provide individualized assessments of risk, they are routinely faced with a fundamental choice: present information that suggests that risks are universal across all individuals in the community or emphasize distinctions about the relative risks across particular groups. The latter is often a preferred strategy among public health officials, in particular because it is thought to support to more efficient allocation of prevention resources to the subsets of the population where they are most needed.

The present research asks whether it is always a good idea to communicate risks in terms of group differences. Are there contexts in which doing so might link a group to socially stigmatized behavior and other negative outcomes that might have consequences for intergroup conflict and political polarization? Our concerns are rooted in a theoretical appreciation of the social construction of risk perception and the implications of intergroup conflict and social identity theories. More broadly, although many everyday dangers are well characterized by experts and policy-makers, a long line of research demonstrates that how individuals *perceive* risks is far from straightforward: risk perception is not merely a function of cold, hard facts concerning the prevalence of certain dangers within a population. In particular, as several scholars have convincingly explained, perceptions of and responses to “objective” dangers tend to deviate from economic notions of expected utility [1–4].

In this article we consider the potential drivers of group-based disparities in risk perceptions and risk-related policy preferences through analysis of a survey experiment of a racially stratified sample of Americans that treats individuals with varying informational frames. Specifically, we focus on attitudes and preferences regarding Acquired Immune Deficiency Syndrome (AIDS) and diabetes. Beginning with the seminal contribution of [5], scholars have demonstrated important *framing* effects on risk perceptions. Informational frames provide alternative perspectives akin to different “visual scene(s)” for understanding the problem about one must make a decision [5]. [6] distinguishes among different types of framing effects. On the one hand, scholars have routinely employed the types of “equivalency” frames discussed in [5], in which the same pieces of information are described in slightly different ways, often varying in terms of positive and negative portrayals. On the other hand, “emphasis” framing involves treating individuals with a different “subset of potentially relevant considerations” [6], i.e. actually providing different pieces of information.

We focus here on the differential impact of emphasis frames with respect to a set of disease threats. In particular, we investigate the effects of information about racial prevalence and about non-normative behavioral risk factors (which we also refer to as blameworthy or stigmatized behaviors). In both cases, the frames were chosen because they provide salient information for risk calculation that could lead individuals to rationally update their risk perceptions. Further, they are both frames that are routinely employed in actual public health campaigns and public discourse around disease threats. Importantly, however, these two frames are not *merely* informative. That is, in addition to providing information relevant to risk assessment, these frames, singly or in combination, potentially invoke intergroup concerns as well as other identity-relevant aspects of information processing. For example, racial prevalence frames may evoke social identity concerns, with citizens internalizing messages about whether their group is at relatively low or relatively high risk. Frames emphasizing non-normative behavioral risk factors link the disease to stigma [7], which individuals might be motivated to minimize or avoid. And combining these two frames by including *both* race prevalence and non-normative behavioral risk factors shifts the stigma from the level of the *individual* to the *group*.

How might these frames affect informational uptake? For members of the lower risk group, such a combined frame might evoke negative stereotypes of the higher risk group, which has now been linked to non-normative behavior via its higher disease-prevalence. On the other hand, for members of the higher risk group, a combined frame might evoke fear of being so stereotyped (a form of social identity threat) or anger that the presentation links negative connotations to their group. Much research in social psychology has documented the psychological power of these linkages in a variety of domains. While the broader pattern of results is somewhat complex, threats to the group can lead to rejection or denial of information and recommitment to the group in the highly identified, and de-identification in those that are not highly identified with the group [8]. Increasing the salience of potentially stigmatizing information at either the individual or group level also frequently leads to disengagement, for example by attributing the information to unfairness or discrimination [9], and effects of this nature have previously been described in the health domain [10, 11]. Thus, on the one hand, our research design allows us to test the sensitivity of individuals to new information that is objectively relevant to risk assessment (Is prevalence higher in my group? Do I engage in behaviors known to be high risk?). On the other hand, we also explore the possibility that the same factors that increase risk assessment on purely objective grounds might in some cases decrease risk assessment when more complex identity and intergroup processes are engaged.

To explore these issues, we proposed and pre-registered several hypotheses related to these concerns. Our hypotheses and analysis plan were pre-registered on April 24, 2014 at e-gap.org, before we analyzed our data. First, when an informational frame emphasizes disparities in disease prevalence across groups, members of those groups will rationally adjust their risk calculations and policy priorities in the direction of the prevalence information. Second, when prevalence information emphasizes non-normative behaviors, individuals will tend to reduce their risk perception as a means of distancing themselves from such behaviors. Third and finally, when both emphases are combined, we expect that identity threat invoked in high-prevalence groups will lead members of that group to distance themselves from the threat by denying the risk, while members of the low-prevalence group will also reduce their risk perception because of the additive effects described in our first two hypotheses.

As we discuss below, even after controlling for a wide range of factors that ought to be clear and proximate predictors of risk perception, we find that both race and gender were strong predictors of risk assessment at baseline. Perhaps in part due to these differences, we do not subsequently find substantial support for our core hypotheses concerning the effects of different emphasis frames introduced in the experimental treatment. While many estimated raw treatment effects are large and in the predicted direction, standard errors are also large, and we do not find many more statistically significant effects than one might expect from chance. Notwithstanding, several of the findings suggest avenues for future research. In particular, informational frames that highlight race disparities positively affect reported risk perceptions and negatively affect reported trust in the government data (that indicates group disparities in prevalence or danger) for members of the high danger group; and we find exactly the opposite for the low danger group. In short, information about racial prevalence can intensify group-based polarization of beliefs.

## 2 Theory and hypotheses

Our central motivation for the study was to better understand the group-oriented or social-relational dynamics of risk perception. While any individual has a potentially unique risk profile given their knowledge, resources, genetics, social networks, and other factors, membership in ascriptively defined groups, such as race or gender, may also independently affect risk perception in contexts where those cleavages are socially salient. In particular, we raise the possibility that group-differentiated messages about particular dangers might have unintended consequences leading to risk denialism. We raise this concern based on a recognition that intergroup relations may strongly affect individual risk perceptions, particularly when a given danger carries negative social connotations—i.e., some form of social stigma, including blame or shame for non-normative behavior. Theoretically, we draw on a series of insights from social psychology [12–14], which emphasize the consequences of intergroup conflict for cognitions and behavior. Of particular relevance for our purposes is the consistent finding associated with social identity theory (SIT) that individuals will use group heuristics to interpret new information, which in turn biases how they process that information as well as their associated attitudes and behaviors. Moreover, individuals strive to develop and to maintain a positive self-image and they will use and protect their group identity to that end [15]. While usually discussed in terms of “self-enhancing” effects such as preferring the ingroup, the same broad motivation should also lead group members to downplay or to deny information that paints their group in a negative light, for example by impugning its reliability or simply not attending to it as fully.

As depicted in Table 1, we develop our propositions with respect to how different types of information affect members of “high-danger” and “low-danger” social groups. Specifically, if information about a given danger is presented in a group-differentiated manner and has been framed (explicitly, or through previous socialization) as the product of potentially blameworthy behavior, individual members of the group identified as being at higher risk are likely to engage in efforts to distance themselves from the threatening portrayal of their group and thereby protect themselves from the shame of association [16]. Although it is theoretically possible for individuals to disidentify, i.e. to distance themselves from the group itself, in the case of salient, personally meaningful, and externally ascribed social identities such as those connected with race and ethnicity, disidentification will often not be a viable strategy. Thus, we anticipate that denialism will be the more common response to this kind of identity threat.

**Table 1:** Treatment conditions and risk groups.

		Treatment arms: (Random assignment) Control	Race-differentiated	Stigmatized	Race-differentiated and Stigmatized
Groups: (Stratified)	Low Danger	LD1	LD2	LD3	LD4
	High Danger	HD1	HD2	HD3	HD4

Following the same logic, we expect that members of the social group identified as being at lower risk of a stigmatized condition (and particularly those individuals who strongly identify with the group) are likely to more positively assess the reliability of the framing information precisely because it deflects the stigmatized risk away from their own group. This also can be interpreted in terms of identity maintenance, in that it also corresponds to perceiving their own risks as being very low.

From this theoretical foundation, our hypotheses pertain to subject populations in which three key conditions hold: there exist identifiable groups within the subject population who engage in a degree of intergroup conflict; there exists a substantial danger which increases in likelihood as a function of presumed voluntaristic/non-normative behavior (e.g., lung cancer and smoking in the American context); and there exists credible data suggesting that the danger is more prevalent in one group as compared with the other. We subsequently refer to these groups as high-danger (HD) and low-danger (LD), respectively. Such conditions are routinely met in the real world, and our examination focuses on two real world health concerns as well as a real world intergroup cleavage, but future research could induce similar conditions in a laboratory context.

Primarily, our research seeks to test the three-way interaction between group identity, the framing of the danger in terms of social group heuristics, and the framing of the danger as the product of stigmatized or “blameworthy” behavior. As such, we consider four different treatment arms being applied to two different groups, for a total of eight treatment conditions:

We advance the three following hypotheses, which grow from our registered pre-analysis plan (in our pre-analysis plan, we specified H2 as our principle research hypothesis and H1 and H3 as auxiliary hypotheses):

- H1: The Group Heuristic hypothesis: In the absence of stigmatization, group-differentiated information will cause individuals to adjust their risk perceptions in line with group membership. Members of the low-danger group should decrease, and those from the high-danger group should increase their risk perception in the case of group-differentiated information about prevalence. With respect to the outcomes of risk perception and support for policies and practices that protect against the specific danger (such as increased budget expenditures or special insurance benefits targeted at that danger), we predict the following relationships post-treatment: LD2 < LD1 and HD2 > HD1.
- H2: The Denialism hypothesis: When high-danger groups are treated with both the stigmatized (blame-worthy) frame and group-differentiated information, we expect to see various manifestations of denialism. In the context of the stigmatized frame, we should see the introduction of information about group-differentiated prevalence lead to a decrease in risk perception (denialism), lower levels of trust in the government data identifying group-differentiated risks, greater reporting of feelings of shame, and less sympathy for those infected, and less support of protective policies among members of the high-danger group. With respect to these outcomes, we predict the following relationship post-treatment: HD4 < HD3.
- H3: The Status Confirming hypothesis: When members of the low-danger group receive both the stigmatizing frame and group-differentiated information, we expect that this information will confirm pre-existing ingroup biases, because this combined treatment implies that members of their own group are at relatively lower risk (as compared with members of the outgroup) for a “blame-worthy” danger. Thus, we expect those from the low-danger group who receive the combined frame will report greater trust in government data identifying group-differentiated prevalence, as compared with low-danger group members who receive only the stigmatized frame. With respect to reported trust in government data, we predict the following relationship post-treatment: LD4 > LD3.

## 3 Methods

### 3.1 Design overview

The crux of the design is a block-randomized framing experiment, which incorporated the four treatment conditions described in Table 1. The experiment was fielded online with a national sample of roughly equal parts white and African-American respondents. Respondents self-reported their race, using a multiple response race question. We coded as white those who self-identified as “White/Caucasian,” including those who identified as some combination of white and some other non-African-American race. We coded as African-American respondents who self-identified as African-American, including those who identified as a combination of African-American and any other race.

All survey data were collected during the summer of 2014 through self-administered online surveys. The majority of responses were collected in a six-week window between late May and early July, but in order to increase the effective sample size of African-American respondents, the researchers collected data from an additional 185 African-American respondents during August 2014. We are comfortable combining the cases because African-American respondents from the early and late summer look similar to one another on demographics and pertinent attitudes about disease. Furthermore, an indicator variable recording when the data were collected is not a significant predictor of attitudes or policy preferences.

Prior to random assignment to treatment condition, subjects were randomly assigned to one of two disease conditions—AIDS or diabetes—which were included so that we could reach conclusions that were not necessarily disease-specific. Randomization of treatment was automated to achieve balance within groups.

Our research protocol was approved by Princeton University’s Institutional Review Board (#5708) as an expedited review because it was deemed to pose only “minimal risk” to participants. At the start of our online survey, we described the nature of the questions and the likely time commitment of participation, and subjects were informed that they could opt out at any time. Subjects were asked about their consent to proceed, and if they clicked affirmatively, the online survey would commence.

All survey participants were invited to participate in two survey waves. The first survey served as a baseline (pre-treatment) survey, primarily gauging demographic characteristics and pretreatment concern about disease. We measured baseline perceptions of risk in such a way as to minimize respondents’ awareness of our particular focus on AIDS and diabetes. The relevant question on the wave one survey asked, “How much of a concern do you think each of the following health and medical problems should be for the future development of the American health care system?” and we offered a sliding response scale with anchors of “Not a concern”; “Minor concern”; “Substantial concern”; “Important concern”; and “Critical concern.” Respondents were asked to select a level of concern for each of the following: asthma, cancer, poor hearing/deafness, diabetes, AIDS, influenza, obesity, poor vision/blindness. Based on this list, we identified a baseline concern with our two key disease conditions. The second wave survey included the experimental treatment, outlined below, and asked about policy preferences, perceived risks, and other outcomes related to public health and health care.

We programmed the survey software to balance random assignment to treatment within blocks of respondents based on responses to the wave 1 survey. Blocks were constructed in terms of self-reported racial identity, gender, income, and pre-treatment responses to questions eliciting concern about diabetes and AIDS. After receiving one of the four treatment conditions, all respondents were asked to respond to questions about policy preferences and perceptions of risk relevant to the particular disease. Because our predictions are distinct for each race group, we analyze the post-treatment data separately for each group and with respect to each disease condition.

This two-wave approach has several advantages. First, by measuring demographics separately from the survey experiment, the design minimizes the chances that questions pertaining to one would contaminate responses to questions about the other. The design is also more efficient than simple randomization because it ensures greater balance of covariates across treatment arms [17–19]. However, this approach was also more expensive than a simple single-wave survey, and involved some attrition of subjects (as discussed below).

### 3.2 Research subjects and data quality

Qualtrics Panels, acting on our behalf, recruited from standing online, non-probability-based panels a national sample roughly-evenly comprised of white and African-American respondents. Prior to fielding the experiment, we conducted a small scale pilot study (N = 100) to gather initial evidence as to effect sizes of interest. This study yielded effect sizes for our hypothesized comparisons ranging from.10 < *f* <.18, and based on our design we prespecified a sample size of 1000 per disease condition to provide us with acceptable power (.89) to detect the smaller of those effects.

Anticipating that many respondents would not return for the second wave, we collected 3,030 total responses in the first wave survey–from 1,255 white and 1,775 African-American respondents–in order to obtain approximately 1,000 white and 1,000 African-American complete cases. Of these respondents, 1,946–including 1,020 self-identified white and 926 self-identified African-American respondents–also completed the second wave. This is to say that we experienced an attrition rate of approximately 36%. Consistent with research on panel attrition (e.g. [20]), we find higher attrition among African-American (48%) than white respondents (19%). Notably, the respondents who returned for the second wave were generally very similar to the larger pool of first wave respondents: the only metric on which they diverge is race—many fewer African-Americans took the second survey. Nevertheless, since there is so little information provided by the respondent in wave 1, we drop from analysis all respondents that failed to participate in the wave 2 survey. Descriptive statistics are presented in Table A in S1 Appendix.

While the data draw on a national pool of respondents, they are not, strictly speaking, nationally representative. By design, our sample includes an oversample of African-Americans. Furthermore, by virtue of the composition of the online pool of subjects from which our sample is drawn, our sample is disproportionately female (59% of respondents are female). Table 2 shows that our sample is not perfectly representative of the U.S. population: compared to the gold-standard Current Population Survey, our respondents are more likely to be female and fewer of our respondents are very poor, have very low levels of education, or are very young. Nevertheless, our use of an online, convenience sample–despite its limitations–is consistent with similar experimental research [21, 22], including research on risk perception (e.g. [23]).

**Table 2:** Comparison of survey subjects and national population.

		Whites		African-Americans	
		Sample	U.S. Pop.	Sample	U.S. Pop.
Gender	Male	43.0%	49.5%	37.9%	46.8%
	Female	57.0%	50.5%	62.1%	53.2%
Age	18 to 24 years	1.7%	11.8%	6.0%	16.0%
	25 to 34 years	13.0%	16.8%	18.8%	19.2%
	35 to 44 years	17.4%	16.2%	21.5%	17.7%
	45 to 54 years	25.2%	18.3%	22.1%	18.7%
	55 to 64 years	28.7%	17.0%	22.6%	15.3%
	65 years and over	14.1%	19.8%	9.1%	13.1%
Education	HS graduate or less	22.8%	41.4%	20.8%	48.6%
	Some college	23.5%	16.5%	31.5%	19.9%
	Associate’s degree	13.1%	10.1%	15.4%	9.6%
	Bachelor’s degree and higher	40.4%	31.9%	32.2%	21.9%
Income	Less than $25K	19.0%	22.7%	25.4%	40.5%
	$25K-$49K	28.8%	25.0%	32.1%	25.7%
	$50K-$74K	20.9%	18.0%	19.1%	15.3%
	$75K-$99K	14.6%	12.0%	11.2%	8.0%
	$100K+	15.6%	22.3%	9.2%	10.4%
	Prefer not to say	1.1%		3.0%

Note: National population figures are derived from the U.S. Census Bureau’s 2014 Current Population Survey

We took precautions not to prime respondents to think about diabetes or AIDS before consenting to participate in the study. Our recruitment materials encouraged panel members to complete a survey on “Priorities in American Health Care.” Our consent script further directed, “Health care in America is an important political issue. Citizens hold many different opinions about which health problems should receive the most attention from government. In this survey, we ask you to share your views about what government should do.” As such, we believe that self-selection bias is minimal because respondents had only a vague notion of the study’s topic when they consented to participate.

We also included several data quality checks in the second, main survey in order to ensure the quality of responses. First, building on [24], we aimed to improve subject attention by training respondents to read carefully. Our training exercise showed all respondents a screen with information about pancreatic cancer and then on the next screen asked the respondents to identify the type of cancer that was previously referenced. This exercise proved relatively easy, as 93% of subjects passed. All respondents continued in the survey except for the 17 subjects (<1%) who failed the training exercise twice and were eliminated from subsequent analysis (these respondents were terminated before the actual experiment, so all following analyses use the reduced sample size of 1,946).

The survey also employed a manipulation check and a more standard attention check. After respondents were randomized to an experimental condition and presented the condition-specific information about diabetes or HIV/AIDS, they were presented two statements and asked which piece of information was presented in the text on the previous screen. In the treatment groups, the correct answer was a statement that summarized the experimental elements. This serves as a manipulation check in the sense that the correct response identifies the elements that are different across the conditions. About 93% of respondents (1,804 of 1,946) passed this manipulation check. Those that failed the manipulation check were shown the control or treatment condition screen again, but were not further quizzed about its content. Finally, the more traditional attention check came in a 10-item matrix near the end of the survey. The last item in the matrix instructed the respondent to “Please select ‘not likely’.” Respondents were not informed whether they passed or failed this attention check. This final check had the highest failure rate of the three types of checks: 13% (259 of 1,946) of respondents either failed to respond to this item or selected some response other than what they were directed to select.

Generally speaking, respondents who failed the manipulation or attention checks exhibit other behaviors suggestive of satisficing or of poor attention to the survey, such as “straight-lining” (repeatedly selecting the same response to items presented in a matrix format) and poor differentiation across presumably opposing items (reporting equal or nearly equal levels of identification with opposing groups like Republicans and Democrats or young and old). Such inattentiveness increases random noise and therefore biases against finding reliable effects; nevertheless, eliminating such cases could limit our generalizability to only attentive respondents. We strike a balance between these poles by preserving inattentive respondents in our main analyses but controlling for inattentiveness with a dummy variable. We also draw attention to instances in which differences across the groups of attentive and inattentive respondents are most pronounced.

### 3.3 Overview of Experimental conditions

Based on respondents’ race, gender, income, and pre-treatment concern about diabetes or AIDS, we randomly assigned white and African-American subjects to one of four experimental conditions for each disease, based on crossing two emphasis frames. The randomization worked such that subjects are balanced across treatment arms on these demographic characteristics (see Table B and C in S1 Appendix). The first focused on the group-based distribution of disease burden and the second focused on non-normative behavioral risk factors. Both diseases were “racialized” by referencing “Black America” and by providing information about the higher rates of the disease among African-Americans. The non-normative behavior treatment reminded respondents that HIV infection can be caused by “unprotected sex with multiple partners” and that people who eat foods “high in sugar and fat” as well as those who do not get adequate exercise have an increased likelihood of getting diabetes. Absent these targeted pieces of information about non-normative behaviors, subjects were told that “researchers continue to study what causes HIV infection” and that “genetics play a role in who develops type II diabetes.” Full screen shots of the entire treatments are available in Figs A-H in S4 Appendix.

Both the racial and behavioral elements are informational emphasis frames because amidst the vast quantity of facts that one might know or learn about a particular problem, and specifically, the problem of the danger of becoming infected with HIV or contracting diabetes, these are two perceived realities that public health officials, the media, and individuals in personal networks may or may not choose to highlight. Both frames have been commonly available in popular discourse and in the media. Crucially, our research depends on our ability to, at least temporarily, affect how individuals come to understand various dangers. If they already hold firm views about these two dimensions, which cannot be even temporarily manipulated, we would not identify any treatment effects.

### 3.4 Outcome measures

Following receipt of the treatment, respondents were asked various questions about how they perceive risks of being infected with cancer and either AIDS or diabetes (depending on which disease condition they were randomly assigned into). The risk perception question asked, “What is the likelihood that the following will be newly afflicted with [CANCER/AIDS/DIABETES] in the next five years? (That is, do not include individuals who already suffer from this disease.)” Respondents were asked to move a slider across a scale that reports to us a quantity from 0 to 100. Respondents did not see the number reported, but rather, they saw seven evenly spaced qualitative anchors, ranging from “No chance,” to “Extremely High.” Respondents were required to consider their own risk as well as the likelihood that “Any member of your family,” “Any close friend,” and “Anyone you know personally” would be afflicted with the particular disease.

Beyond specific questions about risk, we also asked respondents their views on public policy, under the assumption that answers about policy would be a function of risk perceptions. First, we asked, “If you had a say in making up the federal budget this year, should federal spending on each kind of research be decreased, kept about the same, or increased?” And for each of “Cancer Research,” and “[EXPERIMENTAL CONDITION] research,” respondents were asked to provide a response on a 100-point sliding scale with 5 anchors ranging from substantially decreased to substantially increased. We attempt to cross-validate responses to this question by asking respondents to allocate the government’s health budget between just cancer and AIDS or cancer and diabetes, depending on condition. Here, the questions ask, “Imagine you have the opportunity to discuss with your senator how he/she should allocate a portion of the health budget to just these two problems. Assuming that overall effectiveness of prevention methods and treatments are similar on a dollar for dollar basis, how should health spending be allocated? (Total must equal 100%)”. This additional budget question was intended to sidestep the problem that would arise if certain segments of the population favor or oppose all spending increases.

As a final set of policy-related questions, we asked respondents to indicate how much they would be willing to pay for health insurance that would cover advanced diabetes or HIV/AIDS treatment. The question directed respondents to imagine that they were currently paying a $500 monthly premium and then asked how much more they would be willing to pay each month for additional coverage of advanced HIV/AIDS or diabetes treatment. We provided respondents a slider that ranged from $0 to $200. For a baseline, this question also asked about coverage for advanced cancer treatment. (See question wording and distributions in S2 Appendix).

In addition to our primary dependent measures described above, we included several exploratory measures to provide additional insight into the psychological effects of the different emphasis frames. First, to explore the hypothesis that our emphasis frames exert influence in part by cuing emotional responses such as anger, blame, or shame, we also included a set of items asking respondents to report on their emotional responses to learning that a friend had contracted the disease in question. Respondents were asked to respond on 5-point Likert scales reflecting how strongly they would experience each of the following: sympathy, anger, surprise, shame, disgust, worry about the individual’s health, worry about the individual’s friends and family, and wonder about whether the individual had engaged in unsafe or unhealthy behavior.

Second, to explore the possibility that a key mechanism for denialism would be mistrust of information, we ask a question about the respondent’s trust in government data: “How confident are you about the accuracy of official statistics concerning disparities in disease prevalence across RACE groups?” This question was asked in a battery that also asked about confidence in statistics making distinctions by age and gender, to disguise our primary interest in race. It is worth noting that we asked this battery in both the pre-test and in the endline study, which allows us to estimate within- and between-subjects treatment effects. (Specifically, we treat as our dependent variable the difference in reported trust between endline and baseline surveys.)

Third and finally, in order to test whether our experimental manipulations were capable of affecting actual behavior, we concluded the study by offering respondents the opportunity to learn more about their assigned disease. A closing screen announced, “We have no more questions for you, but we would like to provide you an opportunity to learn more about [diabetes/AIDS].” We provided clickable screenshots that were labeled as information about charities, prevention, and testing relevant to diabetes or HIV/AIDS. Unbeknownst to the respondent, we recorded how long the respondent spent with the links and how many total links they clicked on. In the analyses below, we consider a variable that capture the number of items (ranging from 0 to 3) that the individual clicked.

We also included several supplemental measures focusing on whether participants identify with various groups including race, wealth, political, and age-related groups. (For brevity, analyses focusing on these measures are provided in S3 Appendix).

### 3.5 Additional covariates

In order to ensure that random assignment generated relatively comparable groups, we collected data on a number of covariates that arguably should directly affect reported risk perceptions. Specifically, during the baseline survey, we asked respondents to report their gender, age, level of education, and income.

We report summary statistics for these variables separately for African-American and white respondents in Table 3. Additionally, we show in the supplementary materials that the block randomization succeeded such that we have balance in these demographics and in pretreatment concern about disease across the treatment arms.

**Table 3:** Mean pre-treatment concern by race.

	Whites	African Americans	Difference
Concern about cancer	69.3	71.9	2.6*
Concern about HIV/AIDS	50.4	63.4	13.0**
Concern about diabetes	62.5	68.6	6.1**
N	1019	927

*p < .1

**p < .01;

## 4 Analysis and findings

### 4.1 Baseline

In our baseline survey, we asked respondents to identify their level of concern with various health problems on a 0-100 scale, which we interpret as a preliminary measure of risk perception. Table 3 presents average responses across race groups for cancer, HIV/AIDS, and diabetes. We find a substantively large and highly significant relationship between racial identity and risk, with higher risk perceptions among African-American respondents for all three diseases. In further multivariate modeling we find that this race difference persists in the presence of other demographic controls and that gender too plays a role in who is concerned about disease, with women reporting higher estimates of risk (see Table 4). Perhaps the most substantively important finding of our study turns out to be the degree to which African-Americans express higher levels of concern even beyond the large set of factors that distinguish whites and African-Americans in the American context and which we can control for in our sample (i.e., group differences in education, income, residence type, and likelihood of personally knowing someone who is afflicted with one of the diseases discussed in this study.)

**Table 4:** OLS Estimates of Pre-Treatment Concern / Risk Perception.

	Asthma	Cancer	Deafness	Diabetes	HIV	Influenza	Obesity	Blindness
	Model 1	Model 2	Model 3	Model 4	Model 5	Model 6	Model 7	Model 8
Black	10.472***	3.316**	7.148***	7.012***	11.783***	5.006***	6.562***	7.917***
	(1.496)	(1.624)	(1.418)	(1.534)	(1.778)	(1.530)	(1.631)	(1.470)
Female	10.460***	7.673***	6.290***	6.400***	7.003***	6.631***	9.603***	5.391***
	(1.426)	(1.549)	(1.352)	(1.462)	(1.695)	(1.458)	(1.555)	(1.401)
Age	0.169***	0.128**	0.158***	0.171***	0.042	0.213***	0.028	0.295***
	(0.053)	(0.058)	(0.051)	(0.055)	(0.064)	(0.055)	(0.058)	(0.053)
Education	−1.847***	−1.954***	−2.186***	−1.540***	−2.686***	−1.246**	−0.476	−1.991***
	(0.553)	(0.600)	(0.524)	(0.567)	(0.657)	(0.565)	(0.603)	(0.543)
Income	0.489	1.470**	−0.162	1.224*	0.782	0.817	2.242***	−0.529
	(0.635)	(0.689)	(0.602)	(0.651)	(0.754)	(0.649)	(0.692)	(0.624)
Know pers w AIDS	3.113	2.904	1.259	1.049	8.478***	0.661	1.575	2.352
	(1.933)	(2.099)	(1.833)	(1.982)	(2.297)	(1.976)	(2.107)	(1.899)
Know pers w Diab	4.330***	12.741***	3.515**	16.585***	9.816***	6.178***	9.629***	5.593***
	(1.608)	(1.746)	(1.525)	(1.649)	(1.911)	(1.645)	(1.753)	(1.580)
Suburban	−4.585***	−0.164	−1.534	−2.685*	−2.886	−0.609	−0.711	−0.332
	(1.577)	(1.713)	(1.496)	(1.617)	(1.875)	(1.613)	(1.720)	(1.550)
Rural	−1.755	−0.362	−0.784	−0.954	−1.133	2.305	1.493	1.457
	(2.085)	(2.264)	(1.977)	(2.138)	(2.478)	(2.132)	(2.273)	(2.049)
Married	4.497***	1.970	5.756***	2.814*	0.283	2.794*	−0.975	2.518
	(1.567)	(1.701)	(1.485)	(1.606)	(1.862)	(1.602)	(1.708)	(1.539)
Own health past yr	3.357***	3.126***	1.436	4.263***	0.885	2.418**	3.985***	2.423**
	(1.055)	(1.146)	(1.001)	(1.082)	(1.254)	(1.079)	(1.150)	(1.037)
Others’ health past year	3.252***	3.862***	4.403***	3.330***	4.269***	3.751***	3.027***	4.522***
	(1.044)	(1.134)	(0.990)	(1.070)	(1.241)	(1.067)	(1.138)	(1.026)
Discuss health	2.631***	1.918***	2.432***	2.160***	1.634***	2.681***	1.822***	2.600***
	(0.404)	(0.439)	(0.383)	(0.414)	(0.480)	(0.413)	(0.440)	(0.397)
Gay or bisexual	1.158	4.519	3.579	1.734	12.432***	2.221	5.279	1.548
	(3.141)	(3.411)	(2.979)	(3.221)	(3.733)	(3.212)	(3.425)	(3.087)
Constant	1.212	27.924***	11.551***	15.678***	26.791***	7.091	17.845***	11.195**
	(4.579)	(4.972)	(4.342)	(4.695)	(5.442)	(4.682)	(4.991)	(4.499)
N	1864	1864	1864	1864	1864	1864	1864	1864
R-squared	0.133	0.112	0.103	0.158	0.116	0.096	0.098	0.120

*p < .1

**p < .05;

***p <.01;

Each column lists the outcome variable (Concern about respective disease for American health care). Standard errors in parentheses.

Furthermore, while Table 4 shows that race has a substantively large and statistically significant relationship with all eight diseases, the largest relationship by far is with one of our focus diseases, HIV/AIDS. We find that our race dummy variable is associated with an 11.8 point increase on the 0-100 scale, which is approximately one-third of a standard deviation. The effect is also large for diabetes, representing a 7.0 point increase on the same scale. In both instances, we were surprised by the magnitude of these differences after having controlled for many of the factors that differentiate individuals associated with the respective race groups.

We also note consistent relationships with other demographic factors, most notably age and education, with age generally positively related to risk perception and education consistently negatively related to risk perception.

### 4.2 Analysis of treatment effects

Our primary goal was to estimate the treatment effects of our various informational emphasis frames. We analyze the two-wave dataset with ordinary least squares (OLS) regression, including each treatment arm as a separate binary regressor variable, and the control group is left as the omitted category. First, we estimate the treatment effects for each outcome variable in the full dataset (that is, not making distinctions between the two disease conditions), interacting each treatment arm with the race dummy variable, which allows us to estimate the effects for each race group.

$$\begin{array}{c} Y_{i}=\alpha_{i}+\beta_{1}X_{i}^{1}+\beta_{2}X_{i}^{2}+\beta_{3}X_{i}^{3}+\beta_{4}BlackX_{i}^{1}+\beta_{5}BlackX_{i}^{2}+\beta_{6}BlackX_{i}^{3}+\gamma Z_{i}+\epsilon_{i} \end{array}$$

Specifically, *Y*_*i*_ is our outcome measured at *T*_2_. *X*^1^, *X*^2^, and *X*^3^ are dummy variables for the respective treatment arms (race frame only; stigma frame only; race + stigma frame), *Black* is a dummy variable that takes a value of 1 for respondents who self-reported as African-American and 0 for respondents who self-reported as white; *Z* is a vector of pre-treatment covariates (including race and gender) measured at *T*_1_, and *ϵ*_*i*_ is the error term.

**Fig 1:**
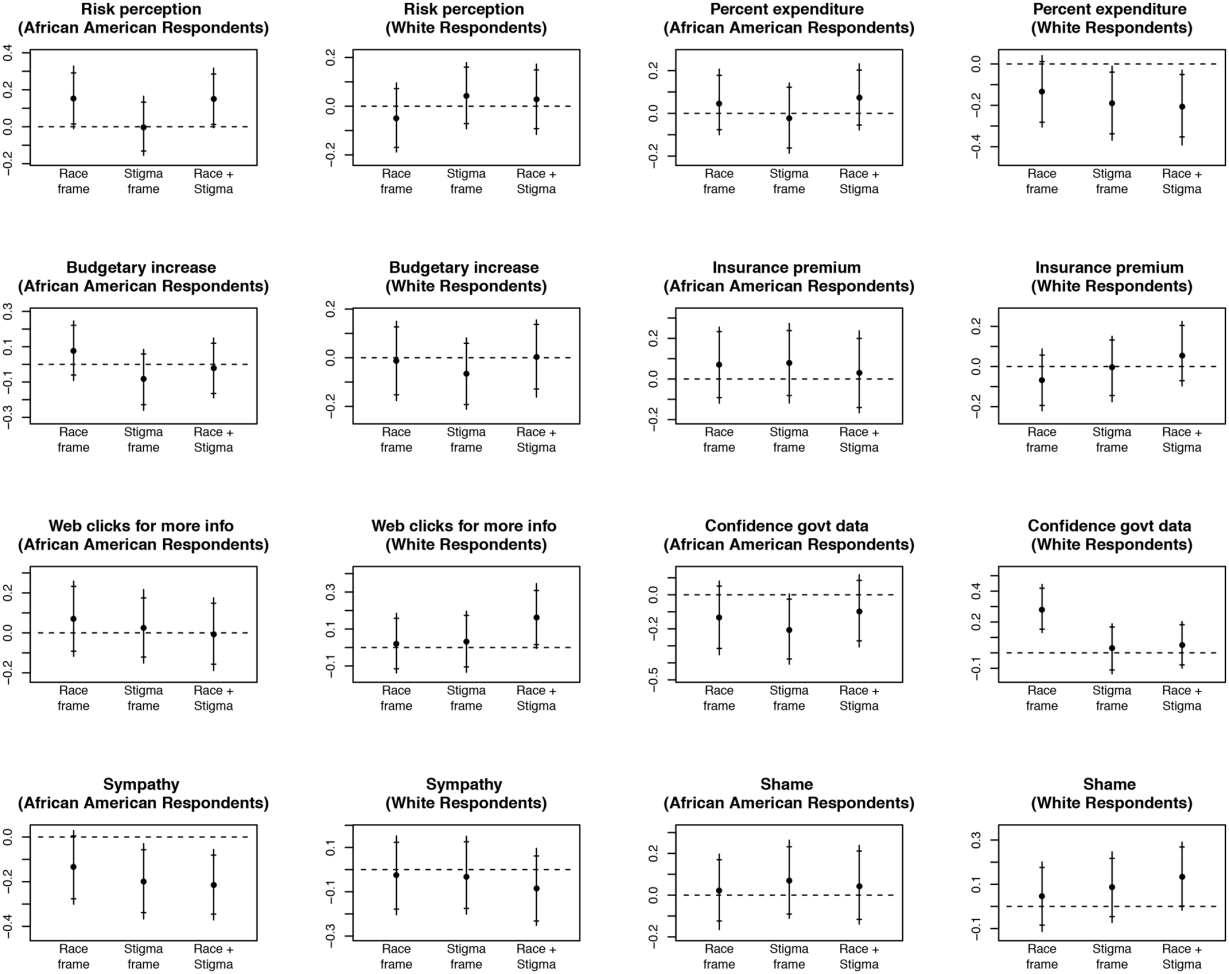
Estimated treatment effects, full sample. Based on sampling 1000 draws with replacement from the observed data, we estimate responses conditional on having received each treatment. Points depict average difference between each treatment condition and control (treatment effect) among members of the indicated respondent group; lines represent 95 percent confidence intervals; horizontal crosses represent 90 percent confidence intervals. Estimated quantities are presented in terms of standard deviations of the outcome variable. Effects are averaged across disease conditions.

After estimating this model, we generate bootstrapped estimates from that regression, calculating the effects of each treatment arm for each of eight outcomes relative to the control condition, first for African-Americans, then for whites (see Fig 1).

Subsequently, in a series of OLS regressions, we estimate the effects of our experimental treatments separately for each disease condition (AIDS or diabetes) and for each race group (African-Americans and whites), which takes the form:

$$\begin{array}{c} Y_{i}=\alpha_{i}+\beta_{1}X_{i}^{1}+\beta_{2}X_{i}^{2}+\beta_{3}X_{i}^{3}+\gamma Z_{i}+\epsilon_{i} \end{array}$$

We report the results from these more disaggregated statistical tests in Tables 5–8. And as we discuss below, while we generally do not find strong support for any of our main hypotheses, we do find some important relationships that merit further investigation. Unless stated otherwise, all effects are estimated relative to the no-treatment control group.

**Table 5:** OLS estimates of Treatment Effects, AIDS Condition (Blacks Only).

	AIDS Risk	AIDS budg shr	AIDS budg inc	AIDS insure	Web clicks	Conf race data (chg)	Sympathy	Shame
	Model 1	Model 2	Model 3	Model 4	Model 5	Model 6	Model 7	Model 8
Treatment: Race only	12.113	3.131*	2.461	−7.263	−0.095	−1.194	−0.291**	0.307*
	(10.259)	(1.846)	(3.035)	(8.620)	(0.135)	(4.709)	(0.116)	(0.159)
Treatment: Stigma only	−4.817	0.479	−3.025	0.898	−0.090	−11.460**	−0.342***	0.246
	(10.369)	(1.876)	(3.068)	(8.675)	(0.136)	(4.770)	(0.117)	(0.161)
Treatment: Race + Stigma	5.089	2.421	−2.316	−3.391	−0.184	−6.251	−0.208*	0.100
	(10.281)	(1.850)	(3.041)	(8.583)	(0.135)	(4.717)	(0.116)	(0.159)
Disease concern	0.143	0.030	0.116***	0.191**	−0.001	−0.040	0.002*	−0.002
	(0.100)	(0.018)	(0.030)	(0.084)	(0.001)	(0.046)	(0.001)	(0.002)
Age	0.028	−0.021	0.143*	−0.004	−0.004	−0.147	0.002	−0.010**
	(0.266)	(0.048)	(0.079)	(0.224)	(0.003)	(0.122)	(0.003)	(0.004)
Female	−4.373	−0.992	1.002	−8.847	−0.098	−5.644	0.021	−0.334***
	(7.776)	(1.403)	(2.300)	(6.512)	(0.102)	(3.576)	(0.088)	(0.120)
Education	0.105	−0.383	−0.318	−1.036	−0.008	0.797	−0.013	0.019
	(2.996)	(0.538)	(0.886)	(2.488)	(0.039)	(1.379)	(0.034)	(0.047)
Income	−3.491	−0.980*	−1.054	1.948	0.028	−2.306	−0.017	−0.064
	(3.185)	(0.572)	(0.942)	(2.659)	(0.042)	(1.464)	(0.036)	(0.049)
Pass manip test	−29.468	−2.880	6.264	−25.242	0.282	11.164	0.358*	−0.168
	(17.978)	(3.229)	(5.319)	(15.732)	(0.237)	(8.213)	(0.202)	(0.277)
Constant	125.537***	47.824***	58.443***	72.725***	0.745**	14.025	4.036***	3.003***
	(24.792)	(4.455)	(7.334)	(21.348)	(0.325)	(11.329)	(0.279)	(0.382)
N	406	403	406	397	408	401	402	402
R-squared	0.023	0.031	0.064	0.027	0.016	0.038	0.042	0.055

*p < .1

**p < .05;

***p < .01;

Each column lists the outcome variable. Treatment arms are dummy variables and the control group is the omitted category. Standard errors in parentheses.

**Table 6:** OLS estimates of Treatment Effects, AIDS Condition (Whites Only).

	AIDS Risk	AIDS budg shr	AIDS budg inc	AIDS insure	Web clicks	Conf race data (chg)	Sympathy	Shame
	Model 1	Model 2	Model 3	Model 4	Model 5	Model 6	Model 7	Model 8
Treatment: Race only	−7.765	−0.616	−2.175	−8.544	0.035	8.246**	0.052	0.109
	(9.127)	(2.291)	(2.866)	(5.895)	(0.112)	(3.320)	(0.131)	(0.140)
Treatment: Stigma only	−16.423*	−2.831	−6.229**	−12.108**	0.003	−5.939*	−0.152	0.197
	(9.161)	(2.292)	(2.865)	(5.963)	(0.112)	(3.314)	(0.131)	(0.140)
Treatment: Race + Stigma	−10.765	−2.532	−4.012	−5.847	−0.052	0.131	−0.139	0.227
	(9.189)	(2.302)	(2.880)	(5.986)	(0.113)	(3.344)	(0.132)	(0.141)
Disease concern	0.411***	0.095***	0.232***	0.266***	0.0005	−0.021	0.009***	0.00002
	(0.091)	(0.023)	(0.029)	(0.059)	(0.001)	(0.033)	(0.001)	(0.001)
Age	−1.001***	−0.103*	0.087	−0.604***	−0.005	0.144	0.002	−0.014***
	(0.243)	(0.061)	(0.076)	(0.156)	(0.003)	(0.088)	(0.003)	(0.004)
Female	−4.955	0.936	1.093	−7.465*	0.124	−2.239	0.331***	−0.160
	(6.526)	(1.637)	(2.044)	(4.211)	(0.080)	(2.376)	(0.094)	(0.100)
Education	2.781	−0.233	0.592	1.260	0.089***	0.832	0.082**	0.013
	(2.548)	(0.637)	(0.796)	(1.649)	(0.031)	(0.925)	(0.036)	(0.039)
Income	−2.307	−1.876***	−1.033	−0.336	0.036	−1.428	−0.001	0.044
	(2.487)	(0.622)	(0.779)	(1.618)	(0.031)	(0.901)	(0.036)	(0.038)
Pass manip test	−24.823	−15.602***	−7.758	−21.052*	−0.214	−0.223	0.020	−0.660***
	(16.727)	(4.156)	(5.222)	(10.859)	(0.188)	(6.011)	(0.238)	(0.254)
Constant	141.815***	56.481***	54.736***	74.069***	0.501*	−1.440	3.039***	2.957***
	(22.763)	(5.674)	(7.114)	(14.654)	(0.270)	(8.206)	(0.325)	(0.347)
N	455	447	451	433	459	444	446	446
R-squared	0.097	0.106	0.149	0.103	0.048	0.056	0.142	0.063

*p < .1

**p < .05;

***p < .01;

Each column lists the outcome variable. Treatment arms are dummy variables and the control group is the omitted category. Standard errors in parentheses.

**Table 7:** OLS estimates of Treatment Effects, Diabetes Condition (Blacks Only).

	Diab Risk	Diab budg shr	Diab budg inc	Diab insure	Web clicks	Conf race data (chg)	Sympathy	Shame
	Model 1	Model 2	Model 3	Model 4	Model 5	Model 6	Model 7	Model 8
Treatment: Race only	14.431	−1.087	0.575	12.692*	0.208*	−5.131	−0.027	−0.159
	(11.381)	(1.678)	(2.739)	(6.694)	(0.111)	(4.028)	(0.115)	(0.134)
Treatment: Stigma only	4.333	−1.145	−1.325	8.398	0.115	−2.499	−0.076	−0.048
	(11.460)	(1.680)	(2.757)	(6.771)	(0.111)	(4.032)	(0.115)	(0.134)
Treatment: Race + Stigma	21.919*	0.141	0.637	6.389	0.128	−0.507	−0.209*	0.002
	(11.376)	(1.681)	(2.742)	(6.714)	(0.111)	(4.033)	(0.115)	(0.134)
Disease concern	0.382***	0.025	0.106***	0.066	0.001	−0.069	0.003***	−0.0001
	(0.124)	(0.018)	(0.030)	(0.073)	(0.001)	(0.044)	(0.001)	(0.001)
Age	0.727**	0.068	0.163**	0.070	−0.005	−0.046	0.005*	−0.013***
	(0.297)	(0.044)	(0.072)	(0.175)	(0.003)	(0.105)	(0.003)	(0.003)
Female	−6.882	−0.470	2.846	−9.639**	0.022	−1.872	0.054	−0.192**
	(8.271)	(1.216)	(1.988)	(4.886)	(0.081)	(2.915)	(0.083)	(0.097)
Education	2.289	0.121	−0.371	−0.115	0.007	−1.919	−0.020	−0.012
	(3.289)	(0.486)	(0.796)	(1.955)	(0.032)	(1.167)	(0.033)	(0.039)
Income	−2.519	−1.198**	1.620*	1.535	−0.023	1.179	0.043	−0.051
	(3.492)	(0.513)	(0.844)	(2.064)	(0.034)	(1.233)	(0.035)	(0.041)
Pass manip test	−4.047	−4.409**	8.833**	−14.297	0.016	−2.773	0.469***	−0.497***
	(14.997)	(2.183)	(3.595)	(8.980)	(0.139)	(5.215)	(0.149)	(0.174)
Constant	129.845***	49.467***	47.873***	59.988***	0.803***	15.689*	3.183***	3.199***
	(26.079)	(3.805)	(6.265)	(15.470)	(0.249)	(9.098)	(0.259)	(0.304)
N	505	494	502	487	519	492	493	493
R-squared	0.043	0.029	0.069	0.027	0.015	0.017	0.060	0.061

*p < .1

**p < .05;

***p < .01;

Each column lists the outcome variable. Treatment arms are dummy variables and the control group is the omitted category. Standard errors in parentheses.

**Table 8:** OLS estimates of Treatment Effects, Diabetes Condition (Whites Only).

	Diab Risk	Diab budg shr	Diab budg inc	Diab insure	Web clicks	Conf race data (chg)	Sympathy	Shame
	Model 1	Model 2	Model 3	Model 4	Model 5	Model 6	Model 7	Model 8
Treatment: Race only	−0.718	−2.699	1.453	1.279	0.025	7.964**	−0.068	0.050
	(9.368)	(1.842)	(2.518)	(6.318)	(0.108)	(3.124)	(0.109)	(0.112)
Treatment: Stigma only	22.937**	−2.236	2.502	11.079*	0.058	5.974*	0.063	0.043
	(9.331)	(1.837)	(2.507)	(6.324)	(0.108)	(3.116)	(0.108)	(0.112)
Treatment: Race + Stigma	16.726*	−2.883	4.375*	11.377*	0.330***	2.470	−0.003	0.096
	(9.370)	(1.842)	(2.518)	(6.343)	(0.108)	(3.131)	(0.109)	(0.112)
Disease concern	0.653***	0.022	0.154***	0.180**	0.001	0.040	0.006***	−0.001
	(0.103)	(0.020)	(0.028)	(0.070)	(0.001)	(0.034)	(0.001)	(0.001)
Age	0.285	−0.001	0.182**	−0.062	−0.002	0.080	0.004	−0.020***
	(0.270)	(0.053)	(0.073)	(0.184)	(0.003)	(0.091)	(0.003)	(0.003)
Female	−10.251	−0.271	0.621	−1.477	−0.265***	−3.027	0.068	−0.075
	(6.785)	(1.331)	(1.821)	(4.583)	(0.078)	(2.261)	(0.079)	(0.081)
Education	−1.466	−0.202	−1.234*	0.350	0.026	−0.726	−0.053*	0.003
	(2.636)	(0.520)	(0.710)	(1.790)	(0.030)	(0.883)	(0.031)	(0.032)
Income	2.094	−0.714	−0.884	7.125***	0.075**	1.238	0.022	0.031
	(2.819)	(0.555)	(0.757)	(1.902)	(0.032)	(0.942)	(0.033)	(0.034)
Pass manip test	−25.964**	−3.589	3.632	−22.981***	0.016	−1.877	0.174	−0.683***
	(11.694)	(2.291)	(3.137)	(7.863)	(0.132)	(3.887)	(0.135)	(0.139)
Constant	136.824***	49.756***	54.071***	34.878**	0.394	−8.079	3.288***	3.179***
	(21.045)	(4.127)	(5.645)	(14.158)	(0.239)	(7.005)	(0.244)	(0.251)
N	557	554	556	550	560	553	554	554
R-squared	0.097	0.016	0.097	0.072	0.063	0.025	0.068	0.141

*p < .1

**p < .05;

***p < .01;

Each column lists the outcome variable. Treatment arms are dummy variables and the control group is the omitted category. Standard errors in parentheses.

We find the most empirical support for hypothesis 1: we expected that the racialized framing treatment would positively affect African-American responses to questions about risk perceptions and protective policies for both disease conditions, and negatively affect white responses. And as depicted in the first plot of the first row of Fig 1, we find that risk perception does increase substantially for African-Americans, and decreases modestly for whites. Moreover, in response to the racialized frame treatment, we find, as expected, modest increases in support for increased budget allocations (for the relevant disease) among African-Americans and *decreases* among whites, though none of these estimates are statistically different from zero. Amongst whites who received the combined racialized-stigma treatment, we estimate a large and statistically significant reduction in preferences for percent expenditure on the given disease (as predicted).

These effects are also estimated in more disaggregated form in the first row of Tables 5–8. We do find that with respect to our total AIDS risk perception measure, the estimated effect for the racialized treatment (only) is relatively large and positive for African-Americans (model 1 in Table 5), and large and negative for whites (model 1 in Table 6), but in both cases, the size of the standard errors is also large enough to limit interpretability. African Americans who received the racialized treatment also reported preferring a larger AIDS budget as compared with those in the control condition, and the reverse was true with respect to whites (models 2 and 3 in Tables 5 and 6), as predicted, but again, these effects were not consistently statistically different from zero. With respect to the question about payment for an extra insurance premium for AIDS, African-American responses were in the opposite direction of our prediction (model 4 in Table 5). And finally, in general, our web click outcomes proved to be noisy measures of risk perception and responsiveness, and we find no significant results with respect to that outcome (model 5 in Tables 5–8).

When considering hypothesis 1 through analysis of data from respondents in the diabetes experiment, the results are also generally directionally consistent with our hypotheses, but certainly not sufficiently precise to reject the null hypothesis of no effect. Although we find that the racialized frame treatment generates substantial *increases* in total risk perception among African-Americans and small *decreases* in risk perception among whites (model 1 in Tables 7 and 8), the estimated effects are again not statistically significant. Moreover, estimated treatment effects with respect to our budget policy questions are not in the predicted direction, nor are they statistically different from zero. One large and statistically significant treatment effect is identified among African Americans (model 4 of Table 7): Those treated with the racialized frame report a willingness to pay a higher insurance premium for diabetes compared with those in the control group (as originally predicted).

With respect to hypothesis 2 (our main hypothesis), we find virtually no empirical support. We had expected that among African-Americans, the combined racialized and stigmatized frame (estimated in row 3 in Tables 5–8) would induce denialism, in turn leading us to observe *negative* point estimates for our central outcomes of interest, and in particular, more negative than with respect to the stigma-only frame. And yet, as clearly depicted in Fig 1, we find just the opposite: the estimated effects of the combined frame are more positive than the stigma-frame alone. With respect to the more disaggregated results among African Americans, we find a positive effect for the combined frame on perceived disease risk (model 1 of Tables 5 and 7), and mixed effects on questions about the budget (models 2 and 3 of Tables 5 and 7), but the only estimate that is statistically significant is in the wrong direction. Moreover, the combined treatment condition had no effect on reported feelings of shame with respect to a friend disclosing their positive HIV status (model 8 of Table 5). And, in all cases, the estimated coefficient in row 3 is more positive than the associated estimated coefficient in row 2, contrary to our stated hypothesis. For example, while the combined frame generated a negative treatment effect for confidence in government data reporting on racial disparities in disease prevalence among African Americans, the treatment effect was also negative but substantially larger and statistically significant in the stigma-only treatment condition (compare rows 2 and 3 of model 6 in Table 5). We do find, as predicted, that the estimated coefficient for the interactive treatment is negative with respect to sympathy in both the AIDS and diabetes conditions. However, only in the case of the diabetes condition, do we find that the estimated coefficient relationship between the combined frame and sympathy is more negative than for the stigma frame alone (compare rows 2 and 3 of model 7 in Table 5).

Finally, with respect to hypothesis 3, the “status-confirming hypothesis,” we do find some empirical support, though not exactly as we had predicted in our pre-analysis plan. Again, we had hypothesized specifically that the interactive treatment would lead to a positive effect—that is, increased confidence in government data reporting such disparities across race groups—among White Americans, the effect sizes are relatively small and they are not statistically different from zero. However, we do find that when whites were presented with the racialized (only) frame, those individuals tended to become significantly more confident in race-based data than those in the control group (see the panel in the fourth column of the third row in Fig 1). Those effects were evident with respect to AIDS (model 6 in Table 6) and diabetes (model 6 in Table 8). By contrast, among African Americans the point estimates of the effects on confidence were negative across all three treatment arms (rows 1–3 of model 6 in both Tables 5 and 7). In short, when White Americans receive information that disease prevalence is worse among African Americans, that generally boosts confidence in those data; and when African Americans receive the same information it diminishes confidence in the data. People seem to place more trust in information that paints their own group in a positive light.

## 5 Discussion

Quite clearly, these data do not provide strong support for our core hypotheses concerning the interactive effects of racialized and blameworthy/stigma informational frames, and in line with growing social scientific norms to report on “null findings,” we do so here. That said, just as a single experiment with positive results would not conclusively demonstrate the power of a set of claims, this single experiment cannot conclusively rule out the validity of our theory and core hypotheses. We suggest here a few possible factors that may have worked against our ability to identify hypothesized effects.

First and most importantly, we were impressed by the magnitude of the inter-group differences in expressed levels of concern at baseline. This suggests that American citizens in our survey sample had already been widely exposed to information highlighting racial health disparities. And this seems to be not simply a function of racially-endogamous social networks, which would lead to differential exposure to particular diseases. Even when controlling for risk factors and health networks, our findings of strong race-based differences suggest the potential power of race-differentiated messaging. But whatever the reason for the baseline disparities, they create a context in which it is exceedingly difficult to influence our respondents through a light emphasis frame in the context of an online survey experiment, precisely because the frame we were providing was already familiar and thus may have exerted its effect across frames. This provides one potential explanation for the limited effectiveness of the information we provided.

Second, in the context of our diabetes condition, it is not clear that our “blameworthy” treatment text had the intended connotation among respondents, as evidenced by the estimated *positive* coefficients for the effects of the associated treatment arm across race groups (quite large and statistically significant for whites). For example, in our AIDS condition analyses, the “blameworthy” treatment had generally negative effects on risk perception and policy priorities, as we had expected. That emphasis frame likely caused respondents to distance themselves from the possibility of infection. By contrast, it may be the case that with respect to diabetes, a discussion of poor eating habits and low levels of exercise actually caused many individuals to focus on their own vulnerability and that of others close to them due to their engagement in those very behaviors. If this was indeed the case, it would imply that our experimental treatment was not an effective instantiation of “stigma” and so would not serve as a strong test of our prediction concerning stigma’s role in risk perception.

Third, we found significant differences in terms of response outcomes by gender at baseline, and analysis of our results suggest different treatment effects by gender—with somewhat stronger treatment effects among women, but still not statistically significant. Indeed, the baseline findings resonate with a substantial theoretical literature that highlights a “white male” effect in risk perception, in which White males underestimate their own risk ([25–27]), which would imply important differences across race *and* gender. Unfortunately, while we expected gender to be a potentially important confounding variable, and we stratified by gender prior to random assignment of treatments, our experiment was not designed, and is under-powered, to analyze heterogeneous treatment effects by gender (though preliminary results are available upon request).

## 6 Conclusion

In this article, we have provided a theoretical discussion of why citizens, despite all of their individual diversity, often perceive disease and other risks not simply as individuals but as members of social identity groups. From our baseline observational research, we confirm the importance of ascriptive group identities as predictors of risk perceptions. Moreover, additional prompts about racial disparities in disease prevalence delivered experimentally were associated with greater risk perception amongst African-Americans, and widened a gap between African Americans and Whites with respect to trust in official government data. This evidence strongly suggests that “social risk” is an important feature of risk perception and how individuals process information and develop policy preferences.

However, our hypotheses concerning the manifestation of inter-group conflict in the dissemination of public health messages find no solid support in our survey experiment. In particular, we predicted that for members of groups known to be at high risk for conditions associated with non-normative or “stigmatized” qualities, they would be more likely to deny those risks when presented with informational frames that emphasized both the high risk to their group and the stigmatized nature of the condition. The data from our experiment suggest that such emphasis frames did not have such an effect.

While we report these findings because we believe it is important to disseminate information from a pre-registered study, we have also highlighted why the findings overall may lead to false inferences of the “type 2” variety. Future research should address these concerns, potentially with other experimental strategies—with respect to other risk conditions, and perhaps with other modalities than a web-based survey.

## Supporting Information

S1 Appendix Data Overview and Balance Across Treatment Arms. (PDF)

S2 Appendix Select Question Wording and Distributions. (PDF)

S3 Appendix Analyses of Group Identification and Racial Polarization. (PDF)

S4 Appendix Screenshots of Treatment Conditions. (PDF)
