# Supplementary material for: Does Stigmatized Social Risk Lead to Denialism? Results from a Survey Experiment on Race, Risk Perception, and Health Policy in the United States
Source: PLoS One. 2016 Mar 10;11(3):e0147219. doi: 10.1371/journal.pone.0147219 (PMC4786345; doi:10.1371/journal.pone.0147219)
Supplement: S1 Appendix — (PDF) [file pone.0147219.s001.pdf]

Social Identity and Social Risk: S1 Appendix
Data Overview and Balance Across Treatment Arms

Yarrow Dunham*
Evan S. Lieberman†
Steven A. Snell‡

*Department of Psychology, Yale University. Email: yarrow.dunham@yale.edu
†Department of Political Science, Massachusetts Institute of Technology. Email: evanlieb@mit.edu
‡Social Science Research Institute, Duke University. Email: steven.snell@duke.edu

1 Data overview

The full dataset and codebook are available at: https://dataverse.harvard.edu

Table A: Summary statistics

**Variable**	**Mean**	**Std. Dev.**	**Min.**	**Max.**	**N**
Age	48.363	13.641	18	86	1946
Concern: Asthma	42.957	31.625	0	100	1946
Concern: Blindness	53.133	30.828	0	100	1946
Concern: Cancer	70.536	33.947	0	100	1946
Concern: Deafness	43.835	29.519	0	100	1946
Concern: Diabetes	65.397	32.847	0	100	1946
Concern: HIV and AIDS	56.614	37.235	0	100	1946
Concern: Influenza	50.627	31.655	0	100	1946
Concern: Obesity	61.781	33.813	0	100	1946
Frequency discuss health	3.768	1.813	1	7	1946
Know someone with AIDS?	0.170	0.375	0	1	1864
Know someone with Diabetes?	0.693	0.461	0	1	1946
Married	0.455	0.498	0	1	1946
Household income	2.616	1.300	1	5	1946
Education	2.779	1.357	1	5	1946
Passed manipulation check	0.927	0.260	0	1	1946
Female	0.592	0.491	0	1	1946
Gay or bisexual	0.053	0.224	0	1	1946
Health problems past year	2.096	0.695	1	4	1946
Pre-treat: identify Afr Amer	60.163	38.308	0	100	1946
Post-treat: identify Afr Amer	60.211	38.035	0	100	1892
Risk AIDS: Anyone you know	29.190	23.876	0	100	861
Risk AIDS: Family member	19.120	20.283	0	100	861
Risk AIDS: Friend	24.895	21.915	0	100	861
Risk AIDS: You	12.022	18.842	0	100	861
Risk AIDS: Total	85.228	72.753	0	400	861
Risk Diabetes: Anyone you know	53.960	26.905	0	100	1062
Risk Diabetes: Family member	46.123	27.705	0	100	1062
Risk Diabetes: Friend	44.946	24.388	0	100	1062
Risk Diabetes: You	37.794	27.460	0	100	1062
Risk Diabetes: Total	182.824	86.337	0	400	1062
Risk AIDS or Diabetes: Total	139.126	94.020	0	400	1923
Increase AIDS budget	65.804	23.683	0	100	857
Increase Diabetes budget	74.303	22.122	0	100	1058
Percent budget on AIDS	37.138	16.539	0	100	850
Percent budget on Diabetes	44.016	14.389	0	100	1048
Percent budget on AIDS or Diabetes	40.936	15.761	0	100	1898
Info-seeking: Number web clicks	0.672	0.908	0	3	1946
How much to pay: AIDS insur prem	41.670	54.889	0	200	830
How much to pay: Diab insur prem	57.795	53.176	0	200	1037
How much to pay: AIDS or Diab (recode)	50.626	54.522	0	200	1867
Is insurance fair?	49.919	27.989	0	100	1840
Pre-treat: Confidence race data	53.052	24.897	0	100	1946
Post-treat: Confidence race data	54.646	24.273	0	100	1890
Change: Confidence race data	1.497	28.907	-100	100	1890
Feel shame for AIDS/Diab victim	1.801	1.073	1	5	1895
Feel sympathy for AIDS/Diab victim	4.083	0.940	1	5	1895

2 Balance across treatment arms

Based on respondents' race, gender, income, and pre-treatment concern about diabetes or HIV/AIDS, we randomly assigned white and African-American subjects to one of four experimental conditions for each disease. Tables S2 and S3 display the distribution of key demographics across HIV/AIDS and diabetes treatment arms, respectively. With few divergences, each cell is very close to 25%, indicating a balanced distribution of these covariates.

Table B: Demographic balance across HIV/AIDS conditions (row percentages)

	Control	Race-differentiated	Blameworthy	Race-diff. & Blameworthy	N
White	24.8	25.1	25.5	24.6	459
African American	25.3	25.0	24.8	25.0	408
Male	25.4	25.1	24.8	24.8	343
Female	24.8	25.0	25.4	24.8	524
HS graduate or less	28.1	23.6	24.1	24.1	195
Some college	21.9	28.1	22.7	24.1	242
2-year college degree	25.4	26.3	22.8	27.3	114
4-year college degree	27.1	22.4	28.0	25.4	214
Graduate degree	21.6	24.5	29.4	22.4	102
Under $25K	26.1	22.1	28.1	23.6	199
$25-49K	23.7	27.3	23.3	25.7	253
$50-74K	23.8	28.7	23.2	24.3	181
$75-99K	25.2	19.6	26.1	29.0	107
$100K+	28.3	24.2	25.8	21.7	120
Prefer not to say	14.3	28.6	28.6	28.6	7

Table C: Demographic Balance Across Diabetes Conditions (row percentages)

	Control	Race-differentiated	Blameworthy	Race-diff. & Blameworthy	N
White	25.2	25.0	25.0	24.9	560
African American	24.5	25.4	25.2	24.8	519
Male	23.6	26.0	25.6	24.9	450
Female	25.8	24.6	24.8	24.8	629
HS graduate or less	26.8	24.2	26.4	22.5	231
Some college	23.8	23.8	27.2	25.2	290
2-year college degree	25.8	27.0	22.7	24.5	163
4-year college degree	24.8	26.4	23.6	25.2	254
Graduate degree	22.7	25.5	24.1	27.7	141
Under $25K	25.2	26.1	25.2	23.5	230
$25-49K	26.0	23.7	24.9	25.4	338
$50-74K	23.4	24.9	29.2	22.5	209
$75-99K	25.3	26.0	22.6	26.0	146
$100K+	23.4	27.4	21.8	27.4	124
Prefer not to say	21.9	25.0	25.0	28.1	32

As an additional test of balance across treatment arms, we modeled the assignment of treatment arm as a function of the same variables, separately for AIDS and diabetes conditions; none of the included demographics predict assignment to condition, further supporting our conclusion of balance across treatment arms.
