# Supplementary material for: Does Stigmatized Social Risk Lead to Denialism? Results from a Survey Experiment on Race, Risk Perception, and Health Policy in the United States
Source: PLoS One. 2016 Mar 10;11(3):e0147219. doi: 10.1371/journal.pone.0147219 (PMC4786345; doi:10.1371/journal.pone.0147219)
Supplement: S2 Appendix — (PDF) [file pone.0147219.s002.pdf]

Social Identity and Social Risk: S2 Appendix
Select Question Wording and Distributions

Yarrow Dunham*
Evan S. Lieberman†
Steven A. Snell‡

*Department of Psychology, Yale University. Email: yarrow.dunham@yale.edu
†Department of Political Science, Massachusetts Institute of Technology. Email: evanlieb@mit.edu
‡Social Science Research Institute, Duke University. Email: steven.snell@duke.edu

We present here the question wording and response distributions of the survey items that serve as our dependent variables. A full codebook is also stored with our dataset.

Risk perceptions

The risk questions were asked in a similar fashion across the diabetes and AIDS conditions and for the baseline comparison to cancer. Respondents did not see numeric values, but were allowed to place a slider anywhere from "No chance," zero, to "Extremely high," 100. Other anchors included, "Very low," "Low," "Medium," "High," and "Very high." The questions stems and average responses were as follows:

Diabetes What is the likelihood that the following will be newly afflicted with diabetes in the next five years? (That is, do not include individuals who already suffer from this disease.)

- You personally? **Whites:** 35.9 **African-Americans:** 39.9
- Any member of your family? **Whites:** 41.5 **African-Americans:** 51.2
- Any close friend? **Whites:** 45.1 **African-Americans:** 44.7
- Anyone you know personally? **Whites:** 53.5 **African-Americans:** 54.5

AIDS What is the likelihood that the following will be newly afflicted with AIDS in the next five years? (That is, do not include individuals who already suffer from this disease.)

- You personally? **Whites:** 11.5 **African-Americans:** 12.6
- Any member of your family? **Whites:** 15.5 **African-Americans:** 23.2
- Any close friend? **Whites:** 22.0 **African-Americans:** 28.1
- Anyone you know personally? **Whites:** 26.1 **African-Americans:** 32.7

Cancer What is the likelihood that the following will be newly afflicted with cancer in the next five years? (That is, do not include individuals who already suffer from this disease.)

- You personally? **Whites:** 37.0 **African-Americans:** 31.6
- Any member of your family? **Whites:** 41.1 **African-Americans:** 38.8
- Any close friend? **Whites:** 43.3 **African-Americans:** 38.1
- Anyone you know personally? **Whites:** 49.9 **African-Americans:** 42.6

Budgetary increases The first budgetary question asked, "If you had a say in making up the federal budget this year, should federal spending on each kind of research be decreased, kept about the same, or increased?" Respondents did not see numeric values, but were allowed to place a slider anywhere from "Decreased substantially," zero, to "Increased substantially," 100. Other anchors included, "Decreased somewhat," "Kept about the same," and "Increased somewhat." The average values by disease and respondent race were as follows:

Diabetes condition
- Diabetes **Whites:** 71.7 **African-Americans:** 77.2
- Cancer **Whites:** 67.3 **African-Americans:** 73.6

AIDS condition
- AIDS **Whites:** 58.7 **African-Americans:** 73.7
- Cancer **Whites:** 72.5 **African-Americans:** 79.5

Budgetary percent expenditures The second budgetary question was asked as a tradeoff between funding to fight cancer and funding to fight either diabetes or AIDS. The question asked, "Imagine you have the opportunity to discuss with your senator how he/she should allocate a portion of the health budget to just these two problems. Assuming that overall effectiveness of prevention methods and treatments are similar on a dollar for dollar basis, how should health spending be allocated? (Total must equal 100%)" The average values by disease and respondent race were as follows:

Diabetes condition
- Diabetes **Whites:** 42.5% **African-Americans:** 45.7%
- Cancer **Whites:** 57.5% **African-Americans:** 54.3%

AIDS condition
- AIDS **Whites:** 32.3% **African-Americans:** 42.5%
- Cancer **Whites:** 67.7% **African-Americans:** 57.6%

Insurance premiums

In both disease conditions, we asked respondents how much more they would be willing to pay each month to cover advanced treatment for cancer and for diabetes or AIDS. The question asked, "Imagine that you were currently paying $500 / month for health insurance that just covered you as an individual. Imagine also that you could pay for extra coverage to guarantee advanced treatment for particular diseases. How much would you be willing to pay EACH MONTH to guarantee access to advanced treatment for the following?" The average values by disease and respondent race were as follows:

Diabetes condition
- Diabetes **Whites:** $ 55.25 **African-Americans:** $60.67
- Cancer **Whites:** $44.67 **African-Americans:** $56.13

AIDS condition
- AIDS **Whites:** $28.01 **African-Americans:** $56.56
- Cancer **Whites:** $49.70 **African-Americans:** $65.01

Web clicks At the end of the survey, respondents in the diabetes and AIDS conditions each had the opportunity to click on links to get more information about the referenced disease. In both conditions there was a link information about charities, about prevention, and about testing. We recorded how many times the respondent clicked on things on this page. The average values by disease and respondent race were as follows:

- Diabetes condition **Whites:** 2.0 **African-Americans:** 2.0
- Aids condition **Whites:** 1.8 **African-Americans:** 2.0

Confidence in race statistics We also asked respondents how confident they were in statistical differences between races. The question asked, "Public health campaigns often highlight that some groups are affected by particular problems or diseases more than other groups. How much do you TRUST the ACCURACY of those statistics, when describing differences in health outcomes between the following sets of groups (age, race, gender)?" and asked specifically about "Race: statistics report differences between African-American and White." We provided respondents a sliding response scaled that ranged from "Don't trust at all," zero, to "Trust completely," 100. "Trust somewhat" was in the middle. Numeric values were not displayed to the respondent. The average values by disease and respondent race were as follows:

- Diabetes condition **Whites:** 56.0 **African-Americans:** 55.4
- Aids condition **Whites:** 54.7 **African-Americans:** 51.7

Sympathy and shame We also asked respondents how they might respond if a close friend disclosed that they had tested positive for either diabetes or AIDS. The question asked, "Please rate how likely you would be to experience the following emotions if a close friend informed you that they tested positive for [diabetes/HIV, the virus that causes AIDS]?" Among other emotions, the survey probed specifically about sympathy and shame. The provided response options were "Not at all likely," "Not likely," "Somewhat likely," "Very likely,", and "Almost certainly." The distribution of responses by disease and respondent race were as follows:

Diabetes Sympathy

- Not at all likely, **Whites:** 61.6% **African-Americans:** 1.4%
- Not likely, **Whites:** 25.5% **African-Americans:** 2.6%
- Somewhat likely, **Whites:** 5.8% **African-Americans:** 18.9%
- Very likely, **Whites:** 3.8% **African-Americans:** 35.1%
- Almost certainly, **Whites:** 3.4% **African-Americans:** 42.0%

AIDS Sympathy

- Not at all likely, **Whites:** 46.0% **African-Americans:** 0.5%
- Not likely, **Whites:** 32.7% **African-Americans:** 1.5%
- Somewhat likely, **Whites:** 12.3% **African-Americans:** 16.2%
- Very likely, **Whites:** 5.2% **African-Americans:** 29.6%
- Almost certainly, **Whites:** 3.8% **African-Americans:** 52.2%

Diabetes Shame

- Not at all likely, **Whites:** 2.0% **African-Americans:** 58.8%
- Not likely, **Whites:** 3.1% **African-Americans:** 22.5%
- Somewhat likely, **Whites:** 23.3% **African-Americans:** 9.9%
- Very likely, **Whites:** 39.2% **African-Americans:** 5.3%
- Almost certainly, **Whites:** 32.5% **African-Americans:** 3.5%

AIDS Shame

- Not at all likely, **Whites:** 2.9% **African-Americans:**4 0.6%
- Not likely, **Whites:** 4.7% **African-Americans:** 30.9%
- Somewhat likely, **Whites:** 24.2% **African-Americans:** 15.4%
- Very likely, **Whites:** 30.5% **African-Americans:** 8.7%
- Almost certainly, **Whites:** 37.7% **African-Americans:** 4.5%
