# Supplementary material for: Does Stigmatized Social Risk Lead to Denialism? Results from a Survey Experiment on Race, Risk Perception, and Health Policy in the United States
Source: PLoS One. 2016 Mar 10;11(3):e0147219. doi: 10.1371/journal.pone.0147219 (PMC4786345; doi:10.1371/journal.pone.0147219)
Supplement: S3 Appendix — (PDF) [file pone.0147219.s003.pdf]

Social Identity and Social Risk: S3 Appendix
Analyses of Group Identification and Racial Polarization

Yarrow Dunham*
Evan S. Lieberman†
Steven A. Snell‡

*Department of Psychology, Yale University. Email: yarrow.dunham@yale.edu
†Department of Political Science, Massachusetts Institute of Technology. Email: evanlieb@mit.edu
‡Social Science Research Institute, Duke University. Email: steven.snell@duke.edu

1 Group Identification

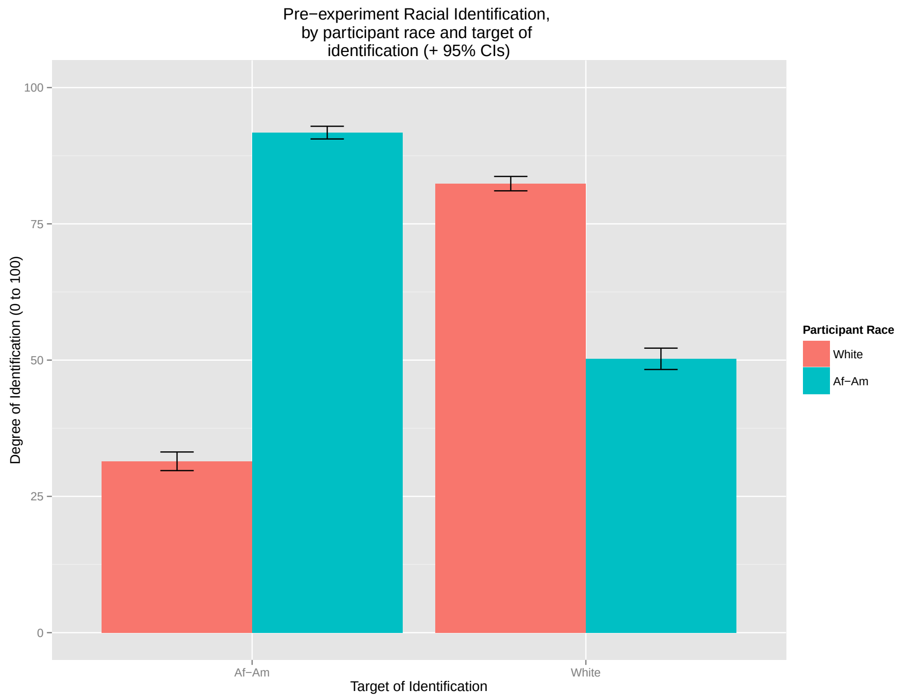

Figure A: Pre-Treatment Racial Identification

A set of eight group identification items were included in both waves of the survey instrument. These were 0-100 sliding scales in which participants indicated their degree of identification with the following: White, African American, rich, poor Republican, Democrat, old, and young. Within-respondent measures of identification across these two time points were reliably correlated, average $r = .63$ (range: .45 to .83, all $p < .001$); given these reliable correlations we focus this summary on pretest Time 1 responses.

Figure A summarizes results of the two racial identification items, sub-divided by participant race. It clearly reveals that racial identification tracked closely with categorical racial identity.

Table 1: Means and standard deviations for group identification items, including effect size(d) and p-value for difference between African American and White respondents

Type of Identity	African American	White	Race difference (d)	p
African American	91.7 (18.1)	31.4 (27.8)	2.54	$<.001$
White	50.2 (30.4)	82.4 (21.4)	-1.23	$<.001$
Democrat	73.6 (28.4)	45.2 (34.2)	.90	$< .001$
Republican	24.7 (26.2)	41.9 (33.3)	-.90	$< .001$
Old	63.5 (30.6)	58.4 (29.0)	.16	$< .001$
Young	62.8 (27.5)	53.7 (26.6)	.34	$< .001$
Rich	25.7 (26.4)	26.5 (24.8)	-.03	$< .58$
Poor	67.0 (28.3)	52.7 (29.1)	.50	$< .001$

Table 1 summarizes responses to all identification items, also sub-divided by race. Further underscoring the divergent identity commitments of African American and white respondents, statistically significant differences are visible on all items except for *rich*, with effect sizes ranging from small to large.

2 Racial Polarization

The second survey wave included four items designed to probe respondent views on racial polarization. Framed in terms of the US context, they asked: whether Whites and African Americans live in separate worlds; whether Whites and African Americans have different needs; whether it's natural for Whites and African Americans to live in distinct communities; and whether it would be desirable to decrease the boundaries between Whites and African Americans. Responses were provided on a 5-point Likert-like scale.

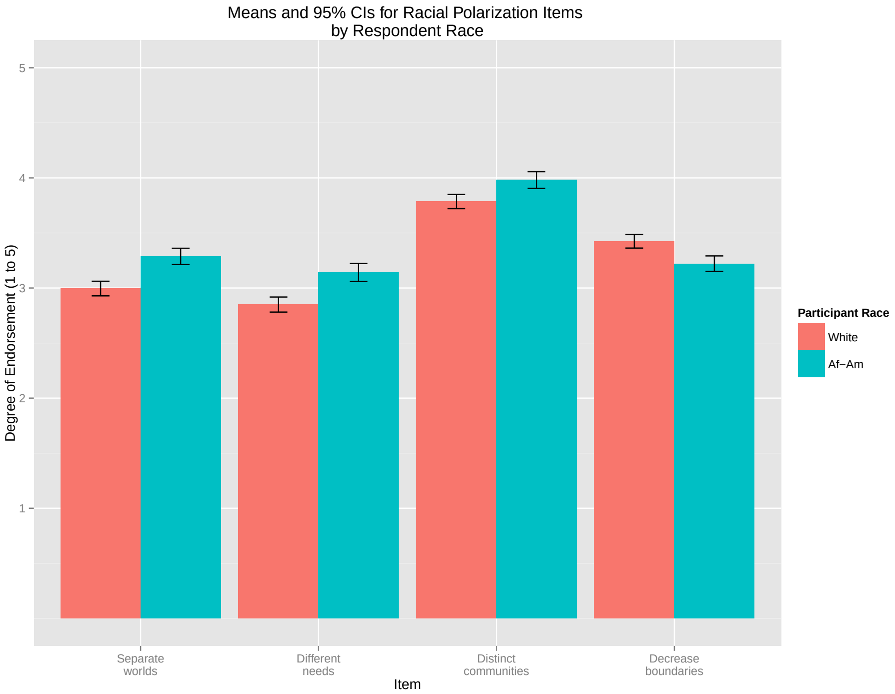

Figure B: Pre-treatment Racial Polarization

Because the items were only weakly correlated ($.04 < r < .39$) we present results for each item separately in Figure B, sub-divided by respondent race. The effect of respondent race was statistically significant in all cases ($p < .001$), with African Americans indicating higher endorsement of the *separate worlds*, *different needs*, and *distinct communities* items, and lower endorsement of the *decrease boundaries* item. However, effect sizes summarizing the magnitude of these differences were small, $.18 < d < .26$.
