# Supplementary material for: Does Stigmatized Social Risk Lead to Denialism? Results from a Survey Experiment on Race, Risk Perception, and Health Policy in the United States
Source: PLoS One. 2016 Mar 10;11(3):e0147219. doi: 10.1371/journal.pone.0147219 (PMC4786345; doi:10.1371/journal.pone.0147219)
Supplement: S4 Appendix — (PDF) [file pone.0147219.s004.pdf]

Social Identity and Social Risk: S4 Appendix
Screenshots of Treatment Conditions

Yarrow Dunham*
Evan S. Lieberman†
Steven A. Snell‡

*Department of Psychology, Yale University. Email: yarrow.dunham@yale.edu
†Department of Political Science, Massachusetts Institute of Technology. Email: evanlieb@mit.edu
‡Social Science Research Institute, Duke University. Email: steven.snell@duke.edu

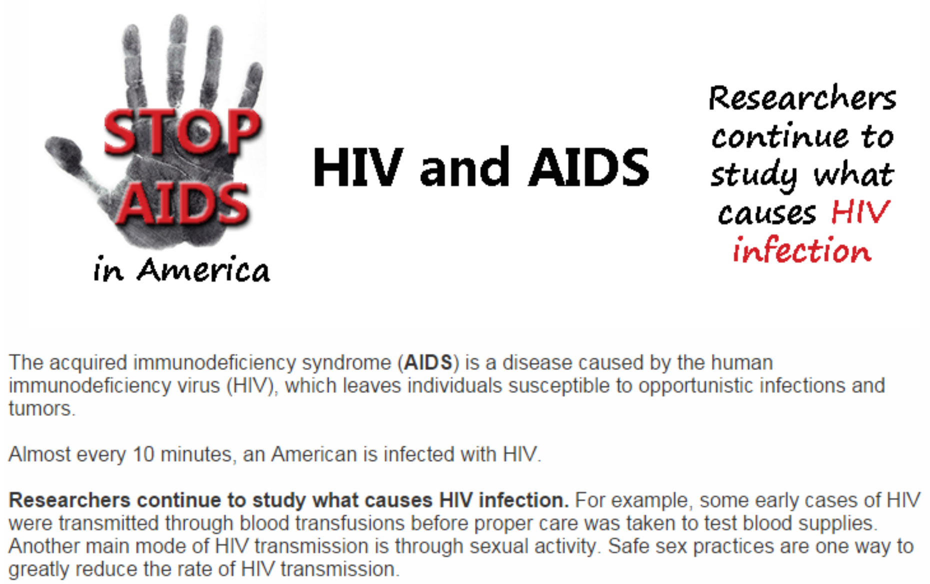

Figure A: HIV/AIDS control condition

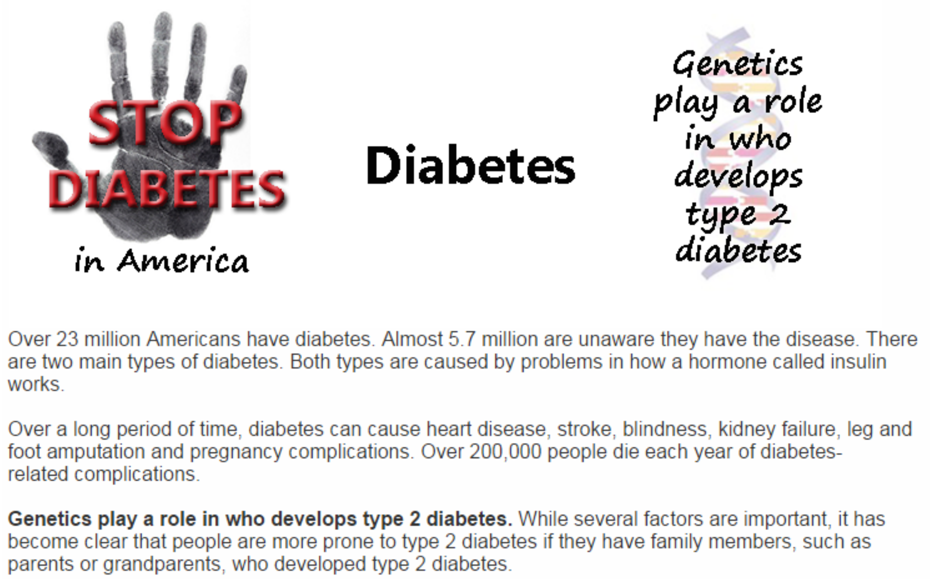

Figure B: Diabetes control condition

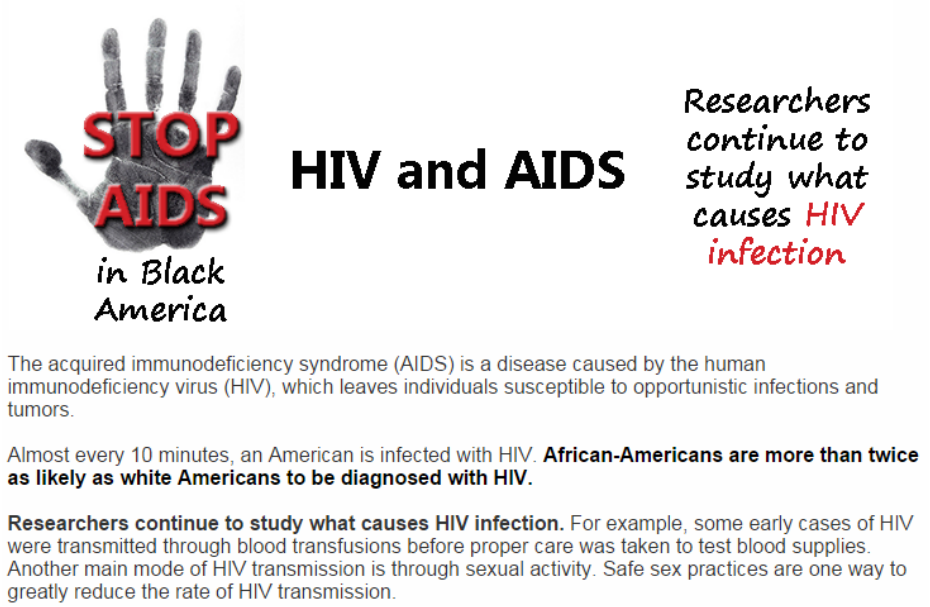

Figure C: HIV/AIDS race-differentiated condition

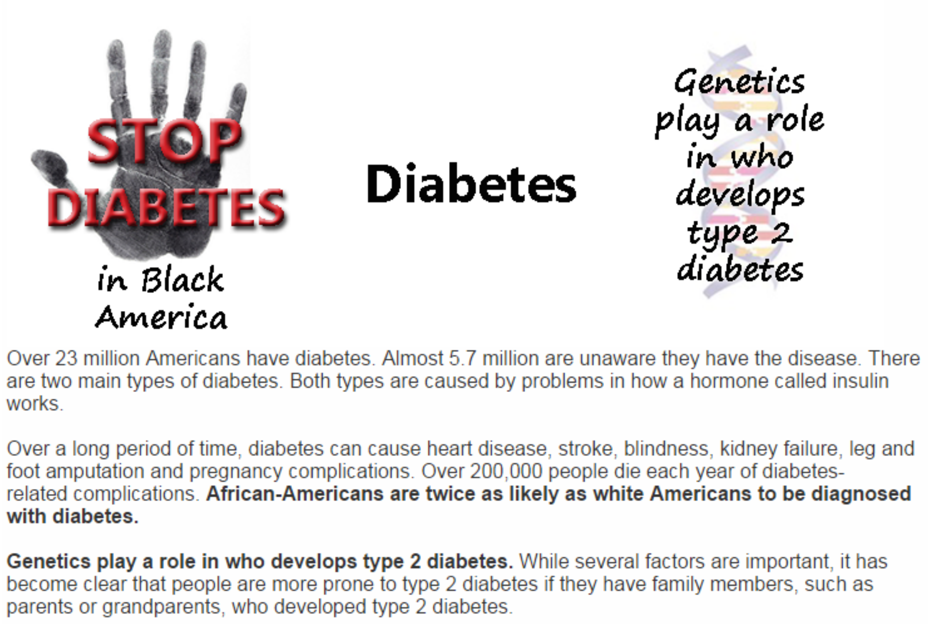

Figure D: Diabetes race-differentiated condition

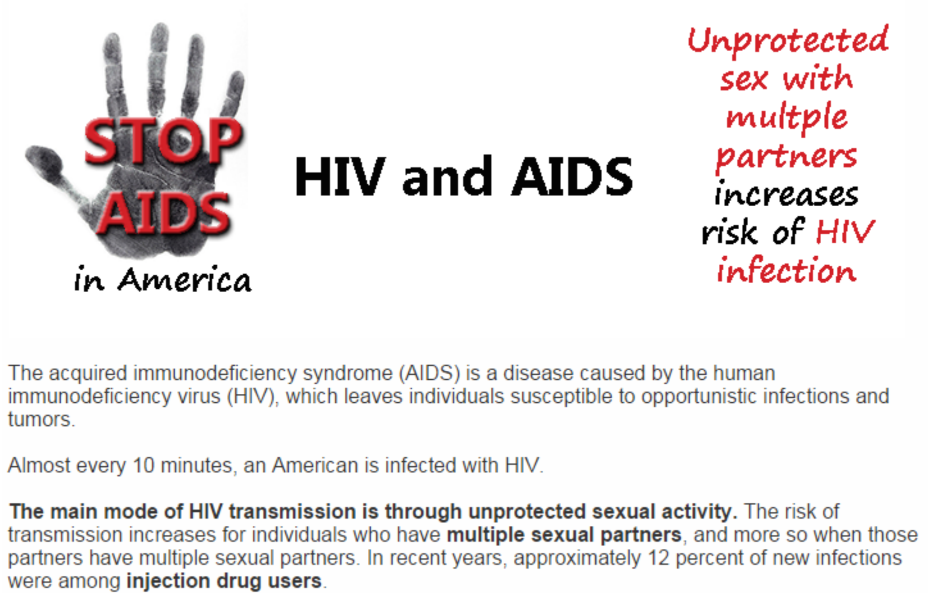

Figure E: HIV/AIDS blameworthy condition

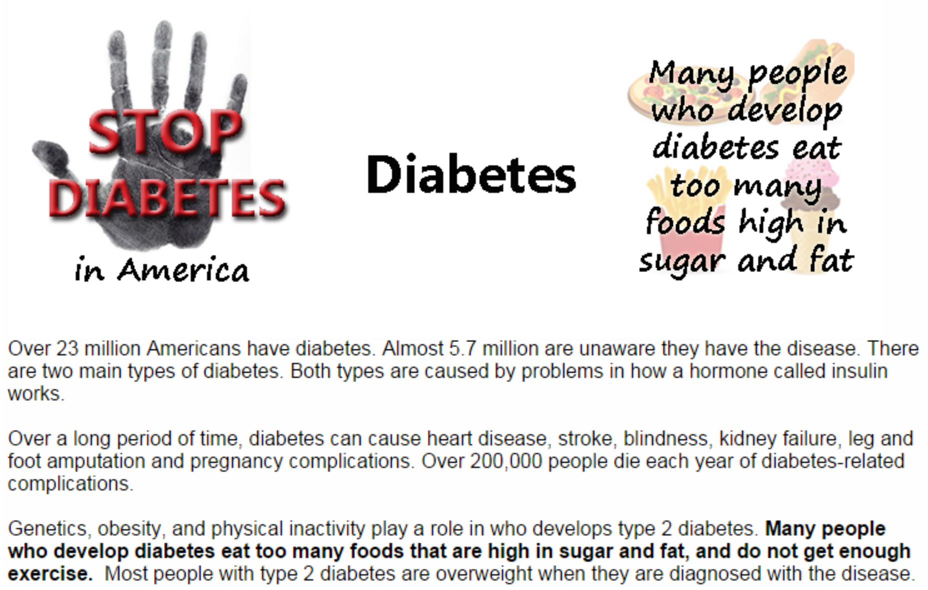

Over 23 million Americans have diabetes. Almost 5.7 million are unaware they have the disease. There are two main types of diabetes. Both types are caused by problems in how a hormone called insulin works.

Over a long period of time, diabetes can cause heart disease, stroke, blindness, kidney failure, leg and foot amputation and pregnancy complications. Over 200,000 people die each year of diabetes-related complications.

Genetics, obesity, and physical inactivity play a role in who develops type 2 diabetes. **Many people who develop diabetes eat too many foods that are high in sugar and fat, and do not get enough exercise.** Most people with type 2 diabetes are overweight when they are diagnosed with the disease.

Figure F: Diabetes blameworthy condition

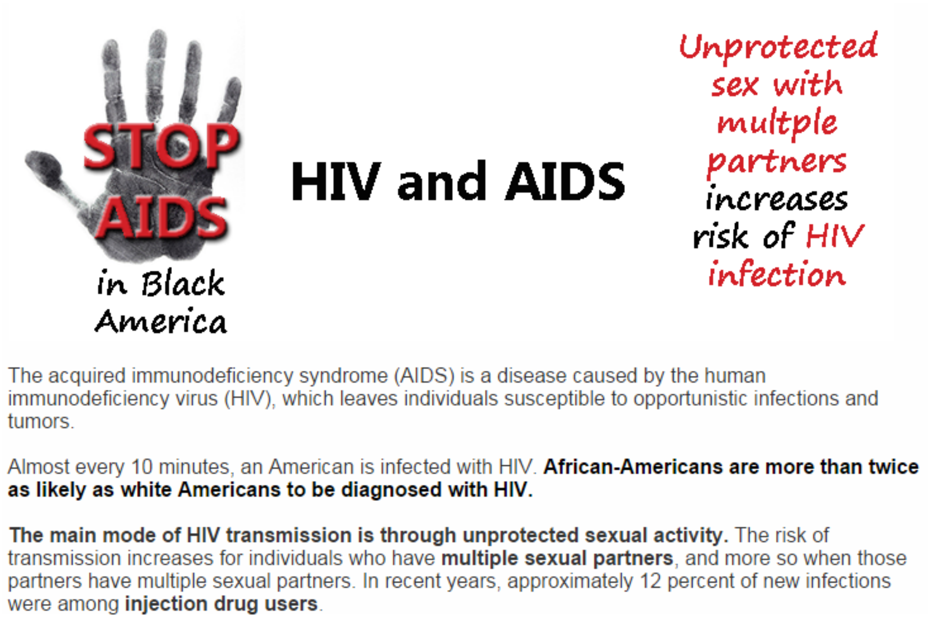

The acquired immunodeficiency syndrome (AIDS) is a disease caused by the human immunodeficiency virus (HIV), which leaves individuals susceptible to opportunistic infections and tumors.

Almost every 10 minutes, an American is infected with HIV. **African-Americans are more than twice as likely as white Americans to be diagnosed with HIV.**

The main mode of HIV transmission is through unprotected sexual activity. The risk of transmission increases for individuals who have **multiple sexual partners**, and more so when those partners have multiple sexual partners. In recent years, approximately 12 percent of new infections were among **injection drug users**.

Figure G: HIV/AIDS race-differentiated and blameworthy condition

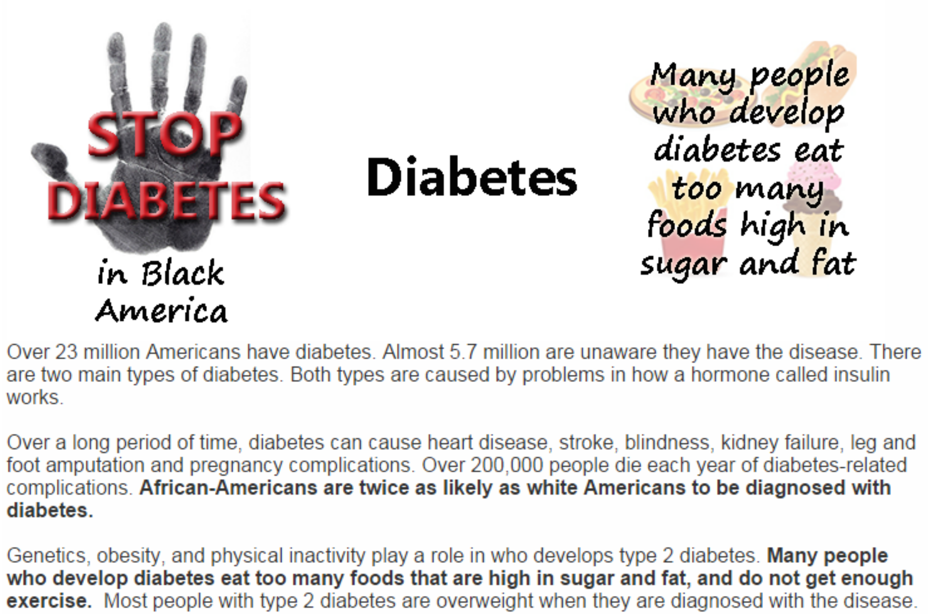

Diabetes

Over 23 million Americans have diabetes. Almost 5.7 million are unaware they have the disease. There are two main types of diabetes. Both types are caused by problems in how a hormone called insulin works.

Over a long period of time, diabetes can cause heart disease, stroke, blindness, kidney failure, leg and foot amputation and pregnancy complications. Over 200,000 people die each year of diabetes-related complications. **African-Americans are twice as likely as white Americans to be diagnosed with diabetes.**

Genetics, obesity, and physical inactivity play a role in who develops type 2 diabetes. **Many people who develop diabetes eat too many foods that are high in sugar and fat, and do not get enough exercise.** Most people with type 2 diabetes are overweight when they are diagnosed with the disease.

Figure H: Diabetes race-differentiated and blameworthy condition
